# *Aloe vera* Adjunctive Therapy for Pediatric Oral Candidiasis: A Prospective Controlled Study on Microbial Clearance and Treatment Adherence

**DOI:** 10.3390/children12111426

**Published:** 2025-10-22

**Authors:** Alexandru-Emilian Flondor, Irina-Georgeta Sufaru, Maria-Alexandra Martu, Vasilica Toma, Stefan-Lucian Burlea, Ioana Martu

**Affiliations:** Faculty of Dental Medicine, “Grigore T. Popa” University of Medicine and Pharmacy, 700115 Iasi, Romaniaioana.martu@umfiasi.ro (I.M.)

**Keywords:** *Aloe vera*, antifungal therapy, colony-forming units, pediatric dentistry, pediatric oral candidiasis, treatment adherence

## Abstract

Background/Objectives: Oral candidiasis is frequently encountered in pediatric populations, particularly in infants and toddlers, where the development of immunity and inconsistent oral hygiene contribute to disease susceptibility. While topical antifungal agents remain the standard of care, treatment challenges persist, especially regarding adherence and recurrence. *Aloe vera*, recognized for its antimicrobial, anti-inflammatory, and mucosal healing properties, may offer therapeutic benefits when used in conjunction with standard regimens. This study aimed to evaluate the adjunctive effect of topical *Aloe vera* gel, when added to standard antifungal therapy, on reducing fungal load and improving treatment adherence in children with moderate oral candidiasis. Methods: A prospective controlled study was conducted among 54 children diagnosed with moderate oral candidiasis. Participants were randomly assigned to receive either standard topical nystatin or nystatin in conjunction with *Aloe vera* gel over a 7 day treatment duration. Fungal load was assessed using colony-forming units (CFU) counts from oral swabs collected at baseline and day 7, analyzed via ANCOVA. Additional parameters included treatment adherence, compared using an independent *t*-test, and clinical recurrence at a 14-day post-treatment follow-up, assessed through logistic regression. Results: Baseline characteristics were similar across groups. By day 7, children in the *Aloe vera* group exhibited a greater reduction in fungal load compared to those receiving standard therapy alone. Adherence was significantly higher in the aloe group (92.73% vs. 89.21%; *p* < 0.0001). Regression analysis identified both baseline fungal burden and adherence as factors associated with an increased risk of recurrence. Conclusions: The addition of *Aloe vera* gel to standard therapy may support a more effective fungal clearance and improved treatment adherence in children with moderate oral candidiasis, suggesting its potential as a complementary treatment option. Given the single-center design, short follow-up, and underpowered recurrence analysis, these findings should be considered preliminary, pending confirmation in larger studies with symptom-anchored endpoints.

## 1. Introduction

Oral candidiasis is a common opportunistic infection in children, especially infants and toddlers, due to immature mucosal immunity and developing oral microbiome [1]. It occurs when host defenses and fungal balance are disrupted, allowing pathogenic overgrowth [2]. *Candida albicans*, a dimorphic fungus, is the leading cause of infection. It usually exists harmlessly but can become invasive under mucosal dysbiosis, immunosuppression, or epithelial damage [3]. Virulence factors like enzymes, biofilm formation, and adherence facilitate this change [4].

Clinically, pediatric oral candidiasis usually appears as pseudomembranous thrush—white, milk curd-like plaques on the buccal mucosa, tongue, and palate that can be wiped away to reveal erythematous, sometimes bleeding mucosa. An erythematous form shows diffuse redness and soreness, often after antibiotic use [5]. Typical symptoms include burning, feeding refusal, irritability, hypersalivation, and dysgeusia, which can impair nutrition and quality of life in young children [6].

Diagnosis is primarily clinical, relying on mucosal lesion appearance. Confirmatory tests like cytology with periodic acid-Schiff (PAS) or Gram staining help visualize hyphae/yeast, especially in moderate or recurrent cases. Culture remains the gold standard for species identification and colony-forming units (CFU) count, using oral swabs on Sabouraud agar or chromogenic media for qualitative and quantitative analysis. Swab sampling is preferred in children due to its noninvasiveness [5,7].

Epidemiologically, pediatric oral candidiasis remains common—especially in low-resource or rural settings where poor oral hygiene, suboptimal nutrition, and limited access to dental care persist [3]. The risk is higher in children with diabetes, immunodeficiency, or prolonged corticosteroid or antibiotic use [8]. In Romania and across Eastern Europe, the condition continues to be a significant reason for pediatric outpatient visits. This is especially true in rural areas, where disparities in healthcare access, dietary quality, and preventive dental care may increase the incidence and recurrence of oral candidiasis among young children [9]. The regional burden emphasizes the need for treatment strategies that are both clinically effective and acceptable to caregivers and children.

Mild cases may resolve without medication in well-nourished children with good oral hygiene [10], but moderate to severe disease usually requires antifungals to prevent progression and reduce symptoms [11]. First-line therapy in pediatrics is topical nystatin or miconazole [12]; these are generally effective [13] but require multi-day adherence [14]. Young children often resist oral treatments (taste, discomfort, sensory issues) [15], so interruptions and inconsistent dosing can lower efficacy [16]. Systemic agents (e.g., fluconazole) are reserved for severe or recurrent cases. Recurrence remains common when mucosal re-epithelialization is incomplete or when predisposing factors (poor hygiene, immunological immaturity) persist [3].

Given these limitations, there is significant interest in adjunct therapies to improve outcomes and patient experience. Non-pharmacological approaches, such as herbal formulations with antimicrobial or anti-inflammatory effects—like *Aloe vera*, tea tree oil, or propolis [17]—and probiotic treatments involving *Lactobacillus* and *Bifidobacterium*, are being studied for restoring oral balance and preventing *Candida* colonization [18]. While their effectiveness remains under investigation, early evidence suggests these may support standard antifungal treatments.

*Aloe vera* shows promise as an adjunct treatment for oral candidiasis: inner-gel extracts exhibit anti-inflammatory, antimicrobial, immunomodulatory, and epithelial-regenerative effects [19]. These effects are driven by acemannan, salicylate derivatives, and polyphenols that suppress pro-inflammatory cytokines, support tissue repair, and regulate oxidative stress in the oral epithelium [20]. Evidence includes in vitro and early clinical research. For instance, Figueiredo et al. demonstrated antimicrobial and anti-inflammatory effects relevant to oral health [21]. Studies report fungal growth inhibition, reduced inflammation, and improved mucosal healing [22]. Its natural origin, safety profile, and ease of application make it well tolerated in pediatric populations [23]. Notably, Rezvaninejad et al. [24] found *Aloe vera* gel inhibited *C. albicans*, *C. glabrata*, and *C. tropicalis* (MIC 312.5–1250 µL/mL), Ahmadi et al. [25] reported larger inhibition zones against *C. albicans* compared to nystatin (*p* < 0.001), and a randomized trial involving adolescents with intellectual disabilities [26] showed an Aloe-containing toothpaste reduced oral *Candida* more effectively than a triclosan-based product. 

Despite the high prevalence of pediatric oral candidiasis and interest in natural adjuncts, clinical evidence for *Aloe vera* gel in children is limited; most studies are in vitro or small adult trials with minimal relevance to pediatrics, few include both clinical and microbiological endpoints, and none evaluate adherence [24,27]. We therefore conducted a study in North-East Romania to determine whether adding topical *Aloe vera* gel to standard nystatin therapy for moderate pediatric oral candidiasis improves symptom resolution, decreases *Candida* CFU burden, and increases adherence. We also sought to explore links between clinical response and compliance. Nystatin, a first-line polyene, was used in both groups to ensure guideline-concordant care. The null hypothesis was that no differences would occur between the aloe adjunct and control groups in clinical severity scores, CFU counts, or adherence rates.

## 2. Materials and Methods

### 2.1. Study Design

This prospective, controlled clinical study was conducted at the pediatric clinic affiliated with “Grigore T. Popa” University in North-East Romania, from February 2024 to May 2025. The study aimed to evaluate the adjunctive effect of topical *Aloe vera* gel in treating moderate oral candidiasis in children. The study was conducted in full accordance with the ethical standards outlined in the Declaration of Helsinki. Informed consent was obtained from the parents or legal guardians of all participants. The protocol received approval from the “Grigore T. Popa” University Ethics Committee (Ethical approval number 390/30.01.2024), and reporting was aligned with applicable principles from the STARD (Standards for Reporting of Diagnostic Accuracy Studies) guidelines to ensure methodological transparency and clarity.

### 2.2. Participants

Fifty-four children, ages 3 to 12 years, clinically diagnosed with moderate oral candidiasis, were enrolled. Participants were recruited from both rural and urban areas across the north-east region of Romania.

#### 2.2.1. Inclusion Criteria

Children were eligible for inclusion if they met the following criteria:

- age between 2 and 8 years;
- clinical diagnosis of moderate oral candidiasis based on a structured severity scoring system (score 5–9);
- availability of informed consent signed by a parent or legal guardian.

#### 2.2.2. Exclusion Criteria

Participants were excluded from the study if they met any of the following conditions:

- prior antifungal therapy–use of systemic or topical antifungal agents within 14 days before study enrollment;
- immunocompromised status;
- chronic systemic illnesses: presence of chronic medical conditions that could alter immune response or mucosal integrity;
- use of immunosuppressive medication;
- severe oral candidiasis: total severity score outside the predefined moderate range;
- other oral pathologies–co-existing oral lesions or mucosal conditions that could confound diagnosis or interfere with outcome assessment (e.g., herpetic stomatitis, geographic tongue);
- known allergy or sensitivity to *Aloe vera* and nystatin.

### 2.3. Randomization and Intervention

Participants were randomly assigned to either the intervention group (nystatin + *Aloe vera*) or the control group (nystatin only) in a 1:1 ratio. A randomization sequence was generated using a computerized random number generator (Microsoft Excel, RAND function) by an investigator not involved in participant recruitment or outcome assessment. Allocation concealment was ensured through the use of sequentially numbered, opaque, sealed envelopes (SNOSE), each containing the assigned group code. These envelopes were prepared in advance and opened only after the participant was enrolled and written informed consent had been obtained from the child’s parent or legal guardian, ensuring that allocation blinding was maintained until the point of intervention assignment.

Although the study was conducted as single-blind, efforts were made to reduce performance and detection bias by blinding the outcome assessors to group assignment. Because of the visible nature of the topical gel, caregivers were not blinded. Participants and their caregivers were only told they would receive standard antifungal treatment, with or without an adjunctive topical gel, but they were not informed about the specific ingredients or expected effects of the adjunctive product. The clinical evaluators and microbiology personnel conducting the CFU analyses remained blinded to group assignments throughout the study.

All participants received topical nystatin oral suspension (100,000 IU/mL) (Mycostatin^®^ Oral Suspension, Substipharm, Paris, France) as the standard antifungal treatment, administered four times daily for 7 days, following current pediatric guidelines for oral candidiasis. The control group (*n* = 27) received nystatin monotherapy.

The Aloe group (*n* = 27) received the same antifungal treatment along with adjunctive topical *Aloe vera* gel. The *Aloe vera* product used was a commercially available preparation containing standardized *Aloe vera* extract (with at least 95% Aloe barbadensis Miller gel content), free from added alcohol and artificial preservatives (Zuccari, Trento, Italy). The gel was applied in a pea-sized amount (approximately 0.5 mL) directly to the affected areas of the oral mucosa using a clean fingertip or sterile cotton applicator, three times daily, after meals and oral hygiene procedures, for 7 days. Caregivers were trained in proper application during the baseline visit. Treatment adherence was assessed using a multi-modal approach. Parents or guardians were instructed to maintain a daily application log, which was reviewed by study personnel at follow-up. Additionally, adherence was cross-validated through direct inquiry and estimation of the volume of remaining medication at Day 7. While self-reported measures inherently carry a risk of reporting bias, this triangulated approach aimed to improve reliability and consistency in adherence estimation.

### 2.4. Study Outcomes

The primary outcome measured in this study was the reduction in fungal load, determined by comparing colony-forming units (CFU) counts obtained from oral swabs collected at baseline and again on the seventh day of treatment. This measure was selected to quantify the microbiological effectiveness of adding topical *Aloe vera* gel to standard antifungal therapy.

Secondary outcomes included (1) treatment adherence, (2) clinical recurrence, and (3) exploratory associations between microbiological or clinical parameters and potential predictive factors. Adherence was monitored through caregiver-maintained daily medication logs, where each administered dose of both the antifungal suspension and topical *Aloe vera* or placebo gel was recorded. Caregivers were instructed to note the timing and completeness of each dose for the 7-day treatment period. These logs were reviewed by study personnel at follow-up visits to ensure accuracy. Adherence was subsequently expressed as a percentage:

Adherence (%) = (Number of doses administered/Total prescribed doses) × 100.

Recurrence was defined as the reappearance of clinical signs or symptoms suggestive of oral candidiasis within 14 days post-treatment, confirmed by clinical examination. Any child presenting with mucosal erythema, white plaques, or symptoms such as oral discomfort and feeding difficulties was assessed for recurrence based on predefined clinical scoring criteria. Recurrence evaluation at day 14 was also performed by a clinician who remained blinded to group allocation.

Additionally, correlational analyses were performed to examine the relationships between adherence, fungal load changes, recurrence, and baseline characteristics such as age and nutritional status, in order to identify potential predictors of treatment response.

### 2.5. Clinical and Microbiological Assessment

Clinical severity was assessed at baseline using a specifically developed structured scoring system. This system aimed to provide a standardized evaluation of oral candidiasis in pediatric patients. It includes five domains: lesion extent, lesion consistency, number of affected areas, pain level, and duration of symptoms. Each domain is scored on a 4-point scale (0–3), allowing a maximum total score of 15 points (Table 1). The scoring framework was adapted from established principles of clinical mucosal assessment tools, such as the Oral Mucositis Assessment Scale (OMAS) [28], and tailored to reflect the characteristic features of candidiasis in young children. The clinical severity scoring system used in this study was previously applied in a study examining oral candidiasis in relation to nutritional status among Romanian children [29]. In that earlier study, the scoring criteria were systematically used across a pediatric cohort, showing consistent alignment with clinical presentation. Although formal psychometric validation has not yet been published, the scale has proven to be a practical and reproducible tool for assessing disease burden in young patients, especially in research involving structured clinical evaluations.

Moderate oral candidiasis was characterized by multiple erythematous or pseudomembranous lesions affecting two or more distinct oral sites (e.g., tongue, palate, buccal mucosa), with or without symptoms such as burning or feeding discomfort, but without any functional impairment. All clinical evaluations were conducted by trained clinicians using consistent scoring criteria.

Oral swabs were collected using sterile cotton-tipped applicators (TD Medical Instruments Co., Ltd., Beijing, China) and immediately placed in sterile transport tubes containing 1 mL of phosphate-buffered saline (PBS). Samples were vortexed for 30 s to ensure even dispersion and then serially diluted from 10^−1^ to 10^−3^. From each dilution, 100 µL was plated on Sabouraud Dextrose Agar (SDA) (Becton, BD Diagnostics, Sparks, MD, USA) supplemented with chloramphenicol and incubated at 37 °C for 48 h in a Heratherm™ Advanced Protocol Microbiological Incubator, Thermo Fisher Scientific, Waltham, MA, USA. Colony-forming units (CFUs) of *Candida* spp. were counted manually, and results were expressed as CFU/mL. The same laboratory personnel and protocols were used for all samples to ensure consistency and minimize inter-rater variability. All microbiological analyses were performed in duplicate by a blinded microbiologist using standardized protocols to ensure reproducibility and minimize observer variability.

Fungal load (CFU/mL) was evaluated solely as a microbiological outcome measure and was not used to classify baseline disease severity, which was based only on clinical criteria. All procedures adhered to standardized protocols for oral microbiological sampling in pediatric populations [6,7].

**Figure 1:**
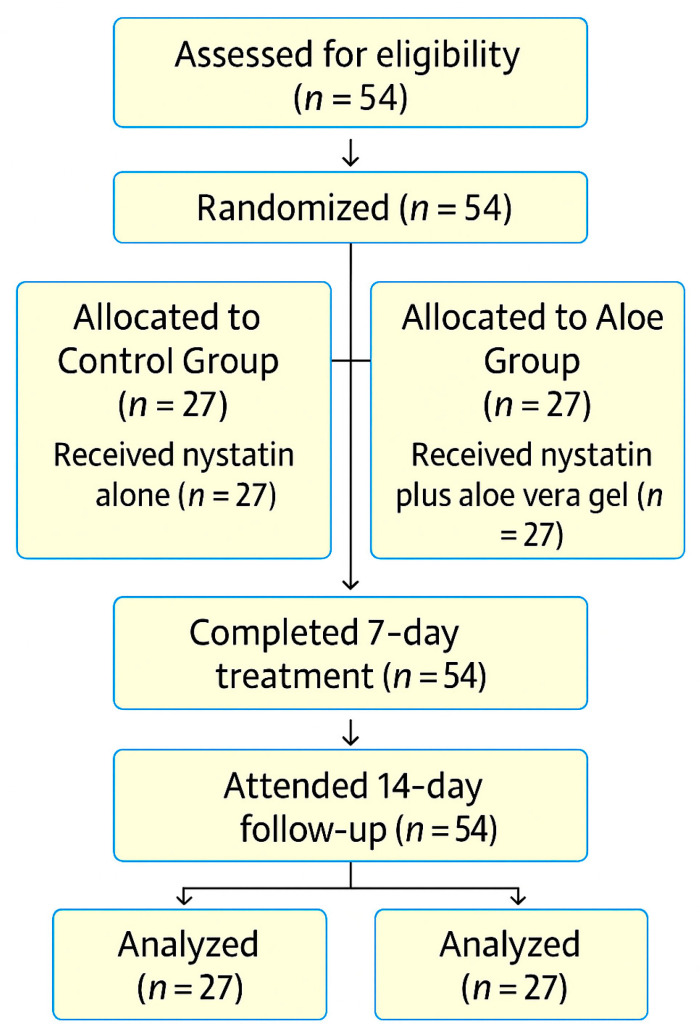
Study flowchart.

The overall progression of participants through the trial—from screening and randomization to treatment allocation, follow-up, and analysis—is summarized in the study flowchart shown in Figure 1.

### 2.6. Sample Size and Power Analysis

The required sample size was calculated using G*Power (version 3.1) based on a pre-study power analysis. The primary endpoint was the reduction in colony-forming units (CFU) at day 7. Assuming a two-sided independent samples *t*-test, with a small-to-moderate effect size (Cohen’s d = 0.5), an alpha level of 0.05, and 80% power, the minimum number of participants needed per group was 27. This results in a total of 54 participants. The effect size was chosen based on previous studies on adjunctive treatments for pediatric oral infections [6] and expected differences in symptom improvement and microbiological response over 7 days. Although the sample size was modest, it was deemed sufficient to detect meaningful differences in microbial clearance and adherence outcomes between groups, supported by observed effect sizes and significance levels during analysis.

### 2.7. Statistical Analysis

All statistical analyses were performed using Python (version 3.10) (Python Software Foundation, Wilmington, DE, USA) and StatsModels (version 0.13.5; StatsModels Developers Team, GitHub, San Francisco, CA, USA). Statistical significance was determined at a two-tailed *p*-value < 0.05. Before inferential testing, the distribution of continuous variables was assessed for normality using a combination of descriptive statistics, histogram plots, and Q-Q plots. Between-group comparisons for normally distributed continuous variables (e.g., age, CFU counts, compliance) were conducted using independent samples *t*-tests. In contrast, categorical variables were compared using chi-square or Fisher’s exact tests, as appropriate.

To assess the primary outcome, CFU at day 7, an analysis of covariance (ANCOVA) was performed, adjusting for baseline CFU values. Adjusted means, standard errors, and corresponding F-statistics were reported.

Effect sizes for inter-group differences in CFU at day 7 and compliance were calculated using Cohen’s d, with standard thresholds applied for interpretation (small: ≥0.2, moderate: ≥0.5, large: ≥0.8). Logistic regression was used to model the probability of recurrence, incorporating treatment group, age, baseline CFU, and compliance as predictors. Odds ratios with 95% confidence intervals were reported. The recurrence model was prespecified as exploratory, and no multiplicity adjustment was applied to secondary outcomes.

Participants were further stratified into high- and low-adherence subgroups based on the median compliance value. In each subset, mean CFU levels were compared between groups using *t*-tests. Correlation analyses (Pearson’s *r*) were also conducted to explore relationships among age, baseline severity, fungal burden, and compliance within each treatment group.

## 3. Results

### 3.1. Demographic Characteristics

A total of fifty-four children diagnosed with moderate oral candidiasis were included in the study, equally distributed between the Aloe and Control groups. All enrolled participants completed the study as planned, with no dropouts or losses to follow-up during the observation period. Furthermore, no adverse reactions were reported among the participants. The average age was 6.89 ± 2.15 years in the Control group and 7.56 ± 1.99 years in the Aloe group. Statistical comparison showed no significant difference in age between the groups (*p* = 0.24) (Table 2).

The gender distribution was well balanced, with males making up 63.0% of the Control group and 44.4% of the Aloe group (*p* = 0.27). Regarding residence, 51.9% of the Control group and 48.1% of the Aloe group were from urban areas, showing no significant difference (*p* = 1.00) (Table 2).

### 3.2. Fungal Load

At baseline, there were no statistically significant differences between the Aloe and Control groups regarding clinical candidiasis severity or fungal burden. The mean baseline CFU was 3166.44 ± 436.29 in the Aloe group and 3126.44 ± 372.91 in the Control group (*p* = 0.71) (Table 3). The baseline severity scores were similarly distributed, with means of 7.22 ± 1.09 and 7.33 ± 1.18, respectively (*p* = 0.71). However, a statistically significant difference in compliance was observed even at baseline, with the Aloe group reporting a mean adherence of 92.73 ± 3.09%, compared to 89.21 ± 2.72% in the Control group (*p* < 0.0001). This difference may reflect early acceptability advantages associated with *Aloe vera* gel.

At day 7, the mean CFU count was lower in the Aloe group compared to the Control group. The unadjusted means were 1089.74 ± 528.87 for the Aloe group and 1316.37 ± 405.18 for the Control group, with a statistically significant difference (*p* = 0.04) based on an independent samples *t*-test.

**Figure 2:**
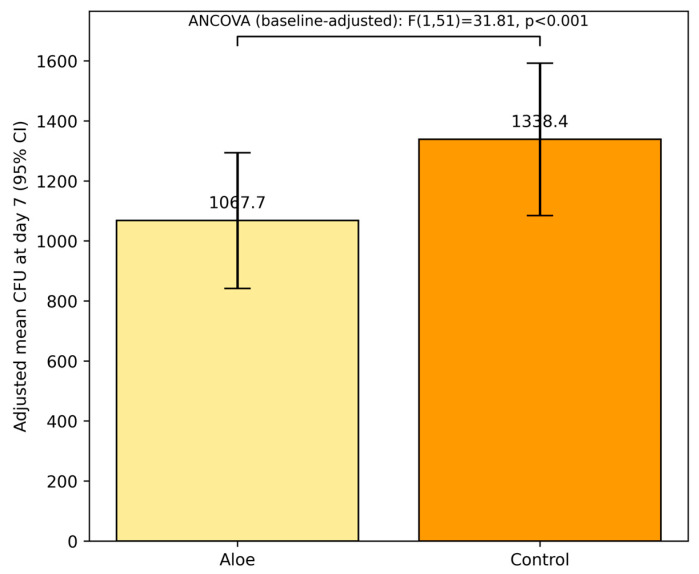
ANCOVA-adjusted mean CFU at day 7 by treatment group (baseline CFU as covariate). Error bars show 95% confidence intervals from model SEs. Group effect: F(1,51) = 31.81, *p* < 0.001; association with baseline: F(1,51) = 478.53, *p* < 0.001.

To account for potential baseline variability, an analysis of covariance (ANCOVA) was conducted using baseline CFU as a covariate. The adjusted model confirmed a highly significant effect of the treatment group on day 7 CFU levels (F(1,51) = 31.81, *p* < 0.001), indicating that the Aloe group maintained a significantly lower fungal burden after adjusting for initial differences in CFU levels (Figure 2). The model also revealed a strong association between baseline and day 7 CFU levels [F(1,51) = 478.53, *p* < 0.001] (Table 4), underscoring the predictive value of initial fungal load on treatment outcome.

### 3.3. Secondary Outcomes

Treatment adherence, measured as percentage compliance, was notably higher in the Aloe group. Participants receiving *Aloe vera* gel had a mean compliance rate of 92.73% ± 3.09, compared to 89.21% ± 2.72 in the Control group (Table 5). This difference was statistically significant (*p* < 0.05), indicating that the Aloe gel was well tolerated and easy to administer, which may have contributed to its superior clinical effect.

### 3.4. Effect Size Analysis

**Figure 3:**
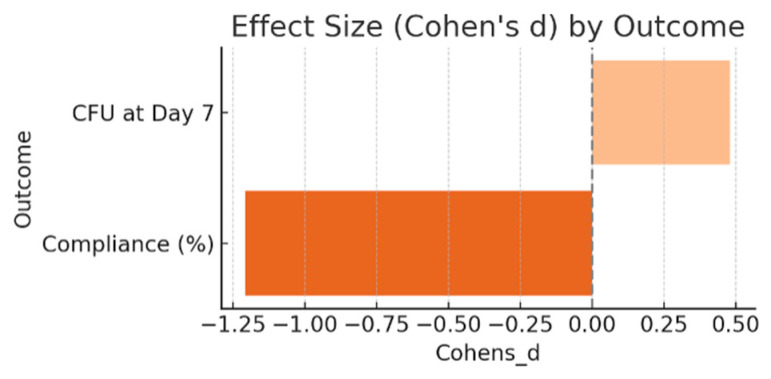
Effect size (Cohen’s d) estimates for key outcomes comparing the *Aloe vera* group to the control group at Day 7. Outcomes include clinical severity score, fungal load (CFU/mL), and treatment adherence. Negative values reflect better outcomes in the aloe group. Benchmarks for small (0.2), medium (0.5), and large (0.8) effects are indicated for reference.

To complement the statistical comparisons, Cohen’s *d* was calculated to estimate the magnitude of group differences for CFU at day 7 and treatment compliance (Figure 3). The effect size for CFU at day 7 between the Control and Aloe groups was 0.48, indicating a moderate difference favoring the Aloe group in terms of fungal reduction. For compliance, the effect size was −1.21, reflecting a significant difference in favor of the Aloe group, where adherence was significantly higher.

### 3.5. Correlation Analysis

**Figure 4:**
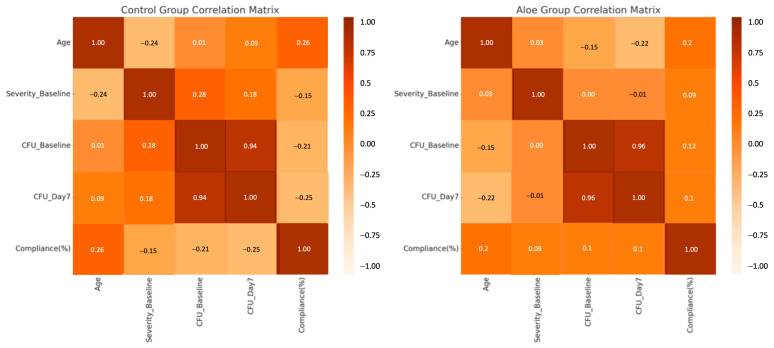
Correlation matrices for the study groups.

Intra-group correlations were analyzed separately for the Control and Aloe groups to better understand the relationships between clinical and microbiological parameters. In the Control group, a strong positive correlation was found between CFU counts at baseline and day 7 (*r* = 0.94), indicating that children with higher initial fungal loads tended to maintain higher levels post-treatment. This trend was similarly observed in the Aloe group, where the correlation between CFU values at the two time points was also high (*r* = 0.96), suggesting consistency in microbial burden across treatment (Figure 4).

Other relationships within the Aloe group were more modest. A weak inverse correlation was noted between age and day 7 CFU levels (*r* = −0.22), while age showed a mild positive relationship with compliance (*r* = 0.20). Notably, in the Control group, compliance was negatively associated with CFU levels on day 7 (*r* = −0.25), although this did not reach statistical significance (Figure 4). These patterns suggest that adherence and initial fungal burden may play a significant role in treatment outcomes, particularly when adjunctive therapies, like *Aloe vera*, are used.

### 3.6. Logistic Regression

A binary logistic regression was used to identify predictors for clinical recurrence after treatment. The variables included were treatment group (Aloe vs. Control), age, baseline CFU count, and compliance percentage.

The analysis identified age as a statistically significant predictor of recurrence (*p* = 0.006), with younger children being more likely to experience recurrence (OR = 0.52, 95% CI: 0.32–0.83). The treatment group also influenced the odds of recurrence, with participants in the Control group showing a higher—but not statistically significant—likelihood of recurrence compared to the Aloe group (OR = 3.83, 95% CI: 0.59–24.99, *p* = 0.159) (Table 6).

Neither adherence (OR = 1.22, 95% CI: 0.91–1.62, *p* = 0.178) nor baseline fungal load (OR = 1.00, 95% CI: 1.00–1.00, *p* = 0.119) reached statistical significance; however, both trended in clinically relevant directions (Table 6). These findings suggest that while age remains a significant factor, the potential impact of adherence and initial fungal burden warrants further investigation in larger, more powered cohorts.

### 3.7. Adherence Stratification Analysis

To further explore the relationship between adherence and treatment outcomes, participants were stratified into high- and low-adherence groups using the median compliance value as a threshold. In the high adherence subgroup, the mean CFU at day 7 was 1139.75 ± 463.08 in the Control group (*n* = 8) and 1197.74 ± 528.16 in the Aloe group (*n* = 19), with no statistically significant difference (*p* = 0.789). However, in the low-adherence subgroup, children in the Aloe group exhibited a substantially lower mean CFU (833.25 ± 463.40, *n* = 8) compared to those in the Control group (1390.74 ± 366.11, *n* = 19), a statistically significant difference (*p* = 0.002) (Table 7).

## 4. Discussion

This prospective, controlled study assessed whether adding topical *Aloe vera* gel to standard antifungal therapy improves microbiological and clinical parameters in children with moderate oral candidiasis. For the primary endpoint, baseline-adjusted Day-7 CFU differed significantly between groups, thus rejecting the null hypothesis for the microbiological outcome. Secondary analyses also favored the aloe group (e.g., better adherence and signs of clinical improvement), although the effects were modest in size. Overall, the data support *Aloe vera* as a potential adjunct—not a standalone therapy—in pediatric oral candidiasis. 

The two groups were similar at baseline regarding age, sex, environment, and initial fungal burden (all *p* > 0.05), supporting valid comparisons between groups. By Day 7, the Aloe group exhibited a lower mean fungal load than controls in the unadjusted comparison (*p* = 0.04). Notably, after adjusting for baseline CFU in ANCOVA, the treatment effect remained significant (*p* < 0.001), indicating a reduction in fungal burden independent of initial load (see Figure 2 and Table 4).

Although the microbiological effect was statistically strong, its clinical significance requires careful interpretation. The adjusted means differed by about 270 CFU/mL, which indicates a small-to-moderate effect size relative to the observed variation. In pediatric oral candidiasis, no validated CFU threshold reliably distinguishes symptomatic improvement, and clinical response is affected by multiple factors (e.g., mucosal healing dynamics and treatment adherence, which was higher in the Aloe group). Overall, these data suggest the microbial change is real but modest, indicating an adjunctive benefit rather than a standalone treatment. Longer follow-up, symptom-based endpoints, and predefined criteria for clinically meaningful change are necessary to determine if this level of reduction consistently leads to noticeable symptom relief.

Several previous studies support our observation of improved fungal reduction with aloe-based treatments [26,30,31]. In a 30-day double-blind trial involving intellectually disabled adolescents, the use of *Aloe vera*-containing toothpaste led to significantly greater reductions in total *Candida* counts—including *C. albicans* and *C. tropicalis*—compared to a triclosan-based control (*p* < 0.05) [26]. Although the study focused on oral hygiene rather than therapeutic use, the results confirm the in vivo antifungal potential of *Aloe vera*.

Controlled laboratory studies also emphasize aloe’s antifungal properties. Rezvaninejad et al. [24] found that *Aloe vera* gel inhibited *C. albicans, C. glabrata*, and *C. tropicalis* in vitro, with minimum inhibitory concentrations (MICs) of 312.5 µL/mL, 1250 µL/mL, and 625 µL/mL, respectively. Similarly, a recent study showed that water-based aloe extracts produced larger inhibition zones against *C. albicans* than standard nystatin (*p* < 0.001) [25]. These in vitro findings support our trial results, demonstrating improved microbial clearance when aloe is used in conjunction with other treatments. They also provide a plausible explanation for the decrease in CFU observed by day 7.

The logistic regression analysis identified age as a significant predictor of recurrence, with younger children at greater risk (OR = 0.52, 95% CI: 0.32–0.83). Although the treatment group variable did not reach statistical significance, the odds ratio (OR = 3.83 for Control vs. Aloe) favored the adjunctive use of *Aloe vera*. Compliance and baseline CFU showed trends toward relevance but did not independently predict recurrence. These findings highlight the multifactorial nature of treatment durability and underscore the importance of individualized approaches in pediatric care.

The absence of recurrence observed in the aloe-treated group during the follow-up period provides valuable clinical insight, particularly in the context of pediatric oral candidiasis, where relapse is common. Recurrence is frequently associated with incomplete mucosal healing, persistent microbial colonization, or suboptimal adherence—factors that conventional monotherapies may not fully address [32]. Although the logistic model for recurrence did not reach traditional significance, the direction and magnitude of the point estimate, along with the baseline-adjusted reduction in Day-7 CFU and higher adherence in the aloe group, suggest a consistent signal of benefit. Considering aloe’s plausible mechanisms—antimicrobial, anti-inflammatory, and pro-healing effects on the oral mucosa—these findings support its credibility as an adjunctive option rather than a standalone therapy. We view the recurrence result as an early protective trend that requires confirmation with longer follow-up and adequately powered, event-driven studies—designs to refine confidence intervals and establish clinically meaningful thresholds.

Treatment adherence emerged as a key factor in therapeutic success. The Aloe group achieved significantly higher compliance rates than the Control group (*p* < 0.0001), suggesting improved tolerability and ease of use. The substantial effect size observed for compliance (Cohen’s *d* = –1.21) further supports the behavioral advantages of *Aloe vera* gel in a pediatric setting. Although the difference in CFU at day 7 yielded a moderate effect size (Cohen’s *d* = 0.48), the observed reduction may carry clinical relevance given the association between fungal burden and persistent or recurrent symptoms.

This study observed a statistically significant but modest 3.5% improvement in treatment adherence with the *Aloe vera* adjunct group compared to the control. Although this numerical difference seems small, it could be clinically meaningful, especially in pediatric populations where adherence to multi-dose regimens often falls short. Even minor increases in adherence can influence treatment effectiveness, particularly for conditions like oral candidiasis that depend on consistent use of topical agents. Importantly, the adherence-stratified analysis showed a significant benefit of *Aloe vera* in children with lower compliance. In this subgroup, the Aloe group had significantly lower day 7 CFU values compared to controls (*p* = 0.0026), suggesting *Aloe vera* may help mitigate the effects of suboptimal adherence. Conversely, no significant differences appeared among children with high adherence, indicating that the additional benefit may be most apparent in real-world settings where achieving perfect compliance is difficult.

The treatment adherence we observed reflects findings in pediatric oral care [25,33,34,35]. In trials involving children aged 8–18, the long-term use of *Aloe vera* mouthwash significantly reduced plaque and gingivitis indices—without the adverse effects often associated with chlorhexidine—and was well accepted by younger users [36]. The sensory properties and ease of application of aloe-based products have been identified as factors that promote higher compliance [37,38,39]. This corresponds with our finding of a large effect size (Cohen’s d = −1.26) favoring the aloe group for adherence.

The improved tolerability and acceptability of *Aloe vera* gel, especially in young children with taste or texture sensitivities, may have contributed to increased compliance. In this study, the topical application of *Aloe vera* gel was well tolerated by all participants in the intervention group. No adverse effects, such as mucosal irritation, hypersensitivity reactions, or gastrointestinal complaints, were reported by caregivers or observed by clinical staff throughout the treatment and follow-up periods. This indicates that using adjunctive therapies that are both effective and child-friendly could be a helpful strategy to improve treatment outcomes in pediatric oral infections. However, long-term safety data in younger age groups remain limited, and future studies should continue to monitor for rare or delayed adverse reactions, particularly when used alongside other medications.

Within the context of existing literature, the observed decrease in fungal load and slightly improved adherence emphasize *Aloe vera*’s potential as an adjunctive agent due to its antimicrobial, anti-inflammatory, and mucosal-soothing properties. These findings agree with earlier studies that demonstrated *Aloe vera*’s inhibitory effects on *Candida albicans* in vitro, while also expanding current understanding by suggesting preliminary clinical benefits in children.

Future research should keep exploring the broader usefulness of integrative strategies in managing oral candidiasis. Specifically, it would be helpful to evaluate the effects of combining *Aloe vera* with other adjunctive methods, such as ozone therapy and photobiomodulation, which have demonstrated clinical benefits in treating periodontal inflammation and promoting mucosal healing [40]. Likewise, probiotics have been examined for their potential to support oral microbial balance and decrease the incidence of dental caries in preschool children, suggesting they may also assist in addressing fungal dysbiosis [41]. Understanding how these interventions might work together could lead to more comprehensive, non-pharmacologic treatments for oral candidiasis, especially in pediatric populations where long-term medication use can present additional challenges.

While this study’s findings significantly improve understanding of adjunctive *Aloe vera* use in pediatric oral candidiasis, several limitations should be recognized. The study population was recruited from a single geographic area, which may limit the generalizability of the results to broader or more diverse pediatric groups. Although the sample size was determined through an a priori power analysis and was deemed sufficient for the primary outcome, the relatively small cohort could still restrict the ability to detect rarer effects or subgroup differences. Therefore, while the preliminary findings indicate that adjunctive *Aloe vera* therapy may enhance fungal reduction, further research with larger cohorts and longer follow-up periods is necessary to verify the robustness and clinical significance of these results.

Another limitation of our study lies in the reliance on caregiver-reported adherence data, which can be affected by social desirability and recall bias. Although we used additional checks, such as estimating medication volume and follow-up interviews, to improve objectivity, future studies could benefit from digital tracking or more quantitative adherence monitoring tools, like medication event monitoring systems (MEMS) or weighed medication tubes.

This study also has a limitation due to its relatively short follow-up period of 14 days. While this duration was enough to observe primary outcomes like clinical improvement and microbial clearance, it might not fully capture the longer-term recurrence patterns often seen in pediatric oral candidiasis. Since recurrence tends to occur within 3–4 weeks, especially in younger children or those with persistent predisposing factors, future studies should consider more extended follow-up periods (e.g., 4–6 weeks) to better evaluate sustained remission and relapse rates. Despite this limitation, our findings still provide valuable preliminary data on early treatment response and adherence patterns.

Although the clinical scoring system used was adapted from previously published models and has been employed in earlier studies, it has not been formally validated through external reliability or construct validity assessments. This represents a methodological limitation and highlights the need for further validation of pediatric-specific tools to assess the severity of oral candidiasis.

Furthermore, microbiological outcomes were evaluated through CFU quantification. However, molecular identification or resistance profiling of *Candida* species was not conducted, which could provide additional insights into treatment effectiveness and pathogen behavior. 

The recurrence analysis was underpowered, as shown by wide 95% CIs around key predictors, a small number of events, and a short follow-up period. Therefore, the data cannot rule out modest protective or adverse effects, and any conclusions about recurrence risk should be considered hypothesis-generating. Larger cohorts with longer follow-up and event-driven power calculations are necessary.

The limitations of this study are similar to those encountered in other pediatric trials investigating adjunctive therapies. For instance, small sample sizes and brief follow-up durations are common in studies on *Aloe vera* or other natural agents used in oral care. This is illustrated by the trial conducted by Khatri et al. [26], which also centered on microbial reduction or adherence but did not include long-term monitoring.

Overall, these findings shed light on the role of adjunctive strategies in pediatric antifungal treatment. By examining both microbiological results and behavioral factors, especially adherence, the study emphasizes the real-world challenges of managing oral candidiasis in young children. The research has several strengths: a prospective, controlled design with balanced randomization; standardized sampling and culture methods; and consistent adherence assessments—all of which strengthen internal validity. Including a rural pediatric group increases relevance to an underserved population, and focusing on a natural product aligns with the rising interest in integrative therapies.

This work contributes to the growing field of phytotherapeutic adjuncts in pediatric oral health. While much of the previous literature focuses on in vitro activity or adult groups, our trial is among the first to assess the clinical effectiveness of topical *Aloe vera* gel as an adjunct to antifungal treatment in children with moderate oral candidiasis. The baseline-adjusted reduction in Day-7 fungal load, combined with improved adherence in the adjunct group, indicates a potential supportive role rather than a standalone treatment.

Importantly, the observed pattern suggests that any benefit of *Aloe vera* may be most noticeable when adherence is less than ideal—a common situation in routine practice. This highlights the importance of evaluating interventions not only for their pharmacologic effects but also for how well they align with patient behavior and real-world use. These findings should be viewed as preliminary and hypothesis-generating; they serve as a foundation for larger, multi-center studies with longer follow-up and symptom-based endpoints to see if modest microbiological improvements lead to consistent, patient-relevant benefits.

## 5. Conclusions

The results of this study suggest that topical *Aloe vera* gel, when used in conjunction with standard antifungal therapy, may offer additional benefits in managing moderate oral candidiasis in children. Adjunctive use of aloe was associated with greater reductions in fungal burden and improved treatment adherence. However, further robust evidence from larger, multi-center cohorts and extended follow-up is required before any clinical recommendations can be made.

## Figures and Tables

**Table 1:** Clinical scoring system for oral candidiasis [29].

Criterion	0 Points	1 Point	2 Points	3 Points
Lesion extent	No lesions	≤25% of oral mucosa	25–50%	>50% or diffuse
Number of areas affected	None	1 area (e.g., tongue)	2 areas	≥3 areas
Lesion consistency	N/A	Easily detachable, thin	Thick, adherent	With erosions/bleeding
Pain/Discomfort	Absent	Mild	Discomfort during feeding	Refusal to feed/crying when nursing
Duration of symptoms	<2 days	2–4 days	5–7 days	>7 days
Interpretation of the Total Score (maximum 15 points): 0–4 points: Mild candidiasis 5–9 points: Moderate candidiasis 10–15 points: Severe candidiasis				

**Table 2:** Demographic characteristics for the study groups.

Variable	Control Group (*n* = 27)	Aloe Group (*n* = 27)	*p*-Value
Age (mean ± standard deviation)	6.89 ± 2.15	7.56 ± 1.99	0.24
Gender (*n*, % Male)	17 (63.0%)	12 (44.4%)	0.27
Environment (*n*, % Urban)	14 (51.9%)	13 (48.1%)	1.00

**Table 3:** Baseline CFU and candidiasis severity by treatment group.

Parameter	Control Group (*n* = 27)	Aloe Group (*n* = 27)	*p*-Value
CFU (mean ± SD)	3126.44 ± 372.91	3166.44 ± 436.29	0.71
Severity (mean ± SD)	7.33 ± 1.18	7.22 ± 1.09	0.71

CFU: Colony-Forming Units; SD: Standard Deviation.

**Table 4:** Analysis of covariance.

Source	df	Sum Sq	Mean Sq	F	*p*-Value
Group	1	693,373.35	693,373.35	31.81	0.0000
CFU_Baseline	1	10,429,215.68	10,429,215.68	478.53	0.0000
Residual	51	1,111,499.79	21,794.11	nan	nan

CFU = Colony-Forming Units; df = degrees of freedom; Sum Sq = sum of squares; Mean Sq = mean square; F = F-statistic.

**Table 5:** Secondary outcomes for the study groups.

Variable	Control Group (*n* = 27)	Aloe Group (*n* = 27)	*p*-Value
Severity Score	7.33 ± 1.18	7.22 ± 1.09	0.719
Compliance (%)	89.21 ± 2.72	92.73 ± 3.09	0.003

**Table 6:** Odds Ratios and Confidence Intervals.

Variable	Odds Ratio	95% CI	*p*-Value
Group [T. Control]	3.83	0.59–24.99	0.159
Age	0.52	0.32–0.83	0.006
CFU_Baseline	1.00	1.00–1.00	0.119
Compliance	1.22	0.91–1.62	0.178

CFU: Colony-Forming Units; CI: Confidence Interval; T: treatment.

**Table 7:** Adherence-stratified CFU outcomes.

Adherence Group	Control Group (CFU ± SD)	Aloe Group (CFU ± SD)	*p*-Value
High Adherence	1139.75 ± 463.08 (*n* = 8)	1197.74 ± 528.16 (*n* = 19)	0.789
Low Adherence	1390.74 ± 366.11 (*n* = 19)	833.25 ± 463.4 (*n* = 8)	0.002

CFU: Colony-Forming Units; SD: Standard Deviation.

## Data Availability

The data used to support the findings of this study are available from the corresponding author upon reasonable request.

## Abbreviations

The following abbreviations are used in this manuscript:

ANCOVA	Analysis of covariance
CFU	Colony-Forming Unit
IU	International Units
OMAS	Oral Mucositis Assessment Scale
